# Cardiac Myxomas Show Elevated Native T1, T2 Relaxation Time and ECV on Parametric CMR

**DOI:** 10.3389/fcvm.2020.602137

**Published:** 2020-11-19

**Authors:** Sarah B. Nasser, Patrick Doeblin, Adelina Doltra, Bernhard Schnackenburg, Katharina Wassilew, Alexander Berger, Rolf Gebker, Tamuna Bigvava, Felix Hennig, Burkert Pieske, Sebastian Kelle

**Affiliations:** ^1^Department of Cardiology, Dar Al Fouad Hospital, Cairo, Egypt; ^2^Department of Internal Medicine/Cardiology, Deutsches Herzzentrum Berlin, Berlin, Germany; ^3^DZHK (German Centre for Cardiovascular Research), Partner Site Berlin, Berlin, Germany; ^4^Institut d'Investigacions Biomèdiques August Pi i Sunyer (IDIBAPS), Cardiovascular Institute, Hospital Clinic, University of Barcelona, Barcelona, Spain; ^5^Clinical Science, Philips Healthcare, Hamburg, Germany; ^6^Department of Pathology, Rigshospitalet, University Hospital of Copenhagen, Copenhagen, Denmark; ^7^Tbilisi Heart and Vascular Clinic, Tbilisi, Georgia; ^8^Department of Cardiothoracic Surgery, Deutsches Herzzentrum Berlin, Berlin, Germany; ^9^Department of Cardiology, Charité University Medicine Berlin, Berlin, Germany

**Keywords:** ECV, T1 mapping, T2 mapping, CMR, cardiac myxoma, magnetic resonance, heart, tumor

## Abstract

**Introduction:** While cardiac tumors are rare, their identification and differentiation has wide clinical implications. Recent cardiac magnetic resonance (CMR) parametric mapping techniques allow for quantitative tissue characterization. Our aim was to examine the range of values encountered in cardiac myxomas in correlation to histological measurements.

**Methods and Results:** Nine patients with histologically proven cardiac myxomas were included. CMR (1.5 Tesla, Philips) including parametric mapping was performed in all patients pre-operatively. All data are reported as mean ± standard deviation. Compared to myocardium, cardiac myxomas demonstrated higher native T1 relaxation times (1,554 ± 192 ms vs. 1,017 ± 58 ms, *p* < 0.001), ECV (46.9 ± 13.0% vs. 27.1 ± 2.6%, *p* = 0.001), and T2 relaxation times (209 ± 120 ms vs. 52 ± 3 ms, *p* = 0.008). Areas with LGE showed higher ECV than areas without (54.3 ± 17.8% vs. 32.7 ± 18.6%, *p* = 0.042), with differences in native T1 relaxation times (1,644 ± 217 ms vs. 1,482 ± 351 ms, *p* = 0.291) and T2 relaxation times (356 ± 236 ms vs. 129 ± 68 ms, *p* = 0.155) not reaching statistical significance.

**Conclusions:** Parametric CMR showed elevated native T1 and T2 relaxation times and ECV values in cardiac myxomas compared to normal myocardium, reflecting an increased interstitial space and fluid content. This might help in the differentiation of cardiac myxomas from other tumor entities.

## Introduction

While cardiac tumors are rare, their identification and differentiation has wide clinical implications. Myxomas represent 25–50% of primary cardiac tumors. They contain variable amounts of stromal and inflammatory cells set in a mucopolysaccharide-rich fibrous matrix (1). Myxomas are mainly localized in the left (60–75% of cases) and right (20–28%) atrium. In rare cases they can affect both atria or the ventricles (2). While echocardiography (trans-thoracic and trans-esophageal) is the first-line imaging modality in the assessment of myxomas, cardiac magnetic resonance (CMR) offers complementary information regarding tissue characterization and distinction of different pathologies (3). Cine images can identify location, size, structure, and involvement of different cardiac and extra-cardiac structures as well as great vessels. Traditional T1 and T2 weighted images (with and without fat suppression) provide qualitative information on tissue fat and free water content relative to normal myocardium (4). T2 weighted images are however prone to slow-flow- and motion-artifacts, varying signal intensity and subjective interpretation (5). To overcome these limitations, T2 relaxation time mapping was suggested to provide a reliable and objective assessment of free water content (6, 7). First pass perfusion assesses vascularity of the tumor (8). Late gadolinium enhancement (LGE) can identify localized areas of fibrosis but performs poorly in diffuse fibrosis, for which T1 relaxation time mapping and ECV measurements provide objective, quantitative information (9, 10). While these parametric mapping methods have been studied thoroughly in myocardial diseases, respective data on cardiac tumor characterization is scarce (11, 12). For myxomas, seven cases with reported T1 relaxation times are available in the literature, of which three also reported T2 relaxation times and one ECV (12). Of these, all showed increased T1 and T2 relaxation times and ECV.

The aim of this study is to examine the range of parametric mapping values of cardiac myxomas and to compare these values with histopathological data.

## Methods

From January 2013 till March 2015, 10 consecutive patients were analyzed. These patients were found to have a mass on trans-thoracic and trans-esophageal echocardiography that was clinically suspected to be a myxoma. Other imaging modalities followed in the preoperative assessment as clinically indicated. CMR was performed for all patients. Patients underwent open heart surgery for removal of the masses and the histopathological examination confirmed the excised cardiac masses to be myxomas in nine cases and fibroma in one, the latter being excluded from further analysis. Case 1 of our study has been previously published as a case report (13). The study complies with the declaration of Helsinki and was approved by the ethics committee of the Charité-Universitätsmedizin Berlin. All examinations have been clinically indicated and patients gave written consent.

### Echocardiography

Trans-thoracic and trans-esophageal echocardiography were performed in all patients. Echocardiography showed a left atrial mass in all patients. Morphological characteristics were suggestive of myxoma (relatively large, pedunculated, mobile mass in the left atrium attached to the inter-atrial septum in most cases except for one where the mass was attached to the posterior mitral leaflet).

### Coronary Angiography

CA was performed in all patients to exclude coexisting ischemic heart disease. One patient showed the presence of a two-vessel disease. There was no evidence of neovascularization of the tumors on angiography.

### CMR Protocol

**Figure 1:**
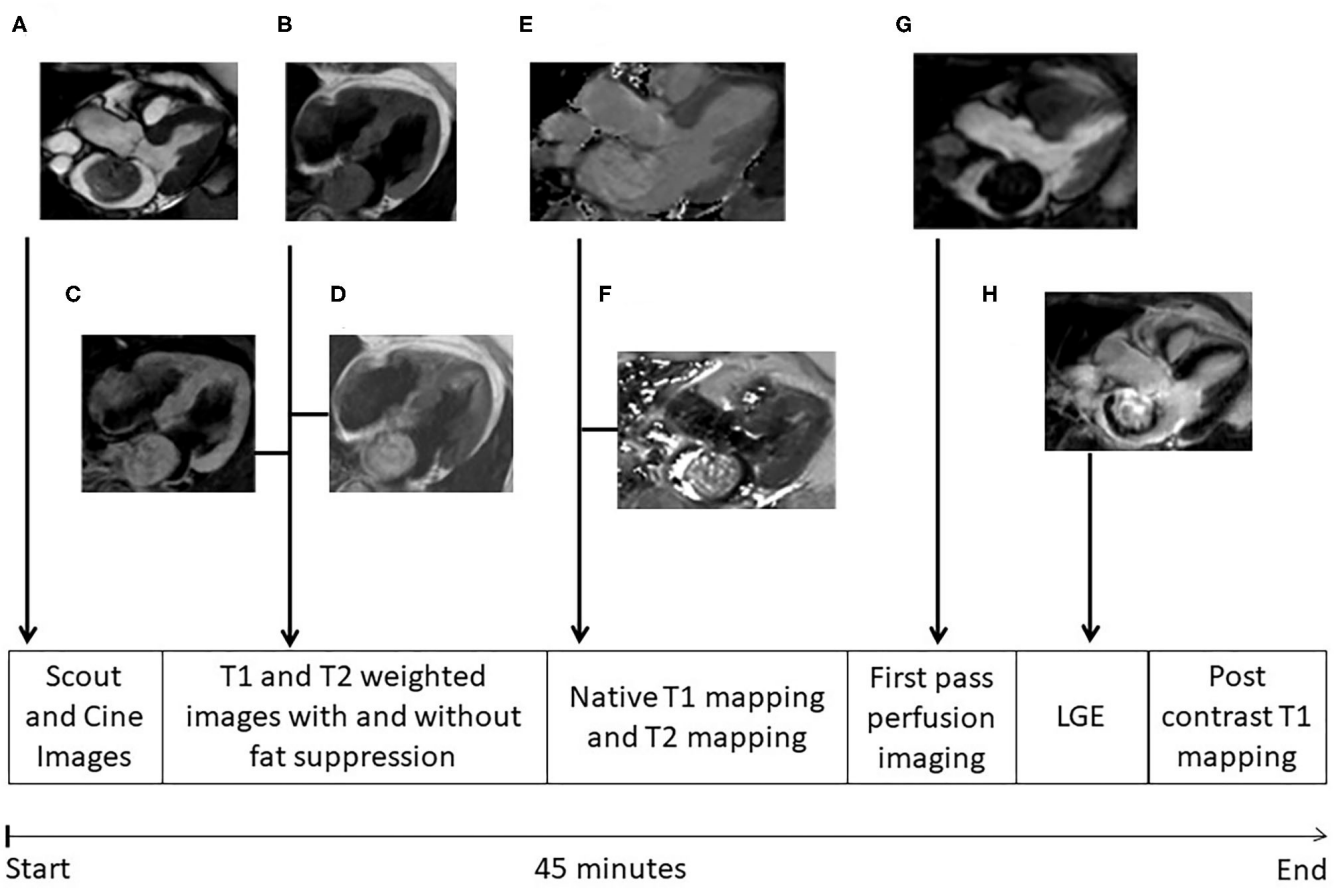
MRI protocol. Different images of cardiac myxomas during cardiac magnetic resonance examination; **(A)** cine image in three chamber view; **(B)** T1 weighted image without fat suppression, myxoma having isointense signal; **(C)** T1 weighted image with fat suppression, myxoma also having isointense signal denoting no fat content in myxoma; **(D)** T2 weighted image without fat suppression, myxoma having hyperintense signal; **(E)** T2 mapping; **(F)** T1 mapping; **(G)** First pass perfusion; **(H)** Late gadolinium enhancement, myxomas showing heterogeneous pattern of delayed enhancement. The whole protocol takes ~45 min to complete.

Cardiac MRI was performed on a 1.5 T scanner (Achieva, Philips Healthcare, Best, The Netherlands). A dedicated 32 channel phased-array surface coil was used as receiver. Sequences were acquired during end-expiratory breath-holds with electrocardiographic (ECG) or pulse triggering. A work-up scheme is given in Figure 1. Standard axial, coronal, and sagittal views were obtained. For functional and anatomical assessment short and long axes cine images were acquired with a balanced gradient-echo cine sequence (spatial resolution 1.8 × 1.8 × 8 mm^3^, 50 heart phases, TR/TE = 3.2/1.6, flip angle 60°) (Figure 1A). T1-weighted turbo spin echo (TR/TE = 1 heart beat/20 ms, voxel size: 1.5 × 1.8 × 8 mm^3^, fat saturation, and black blood pre-pulse) and T2- weighted fast spin echo (TR/TE = 2 heart beats/90 ms, voxel size: 1.4 × 2 × 8 mm^3^, black blood pre-pulse) sequences were performed (Figures 1B–D). First pass perfusion was performed with a balanced steady-state free precession (bSSFP) sequence (TR/TE = 3.2/1.6 ms, flip angle 50°, voxel size: 2.5 × 2.5 × 10 mm^3^) (Figure 1G). Late gadolinium enhancement images after 10–15 min of contrast injection (0.15 mmol/kg gadobenate meglumine, Dotarem, Guerbet, Villepinte, France) was obtained using inversion-recovery 3-dimensional spoiled gradient echo sequence (20 slices, TR/TE = 3.5/1.7 ms, flip angle 15°, voxel size: 1.6 × 1.8 × 5 mm^2^, parallel imaging (SENSE) with an acceleration factor of 2) (Figure 1H). ECG-triggered single-breath-hold Modified Look-Locker Inversion-recovery (MOLLI) sequences were obtained before and 15 min after contrast injection in the planes where the tumor could best be visualized. The sequence consisted of two inversion pulses with different pre-pulse delays (300 and 130 ms), after which the images were acquired following a 5s–(3s)−3s scheme (i.e., Pause interval between the two inversion series was 3s) (Figure 1F). A single shot balanced SSFP was used for signal read out with following parameters: voxel size of 2 × 2 × 10 mm^3^, TR/TE: 2.3/1 ms, flip angle 35°, parallel imaging (SENSE) with an acceleration factor of 2. In addition T2 mapping images were taken. An ECG-triggered breath hold GraSE sequence was used (14) with 9 echoes, EPI factor = 7, TE1 = 9 ms, delta TE = 9 ms, voxel size: 2 × 2 × 10 mm^3^, black blood pre-pulse, parallel imaging (SENSE) with an acceleration factor of 2 and 12 s breath hold time (Figure 1E).

### Quantitative Analysis of T1 and T2 Relaxation Times and ECV

**Figure 2:**
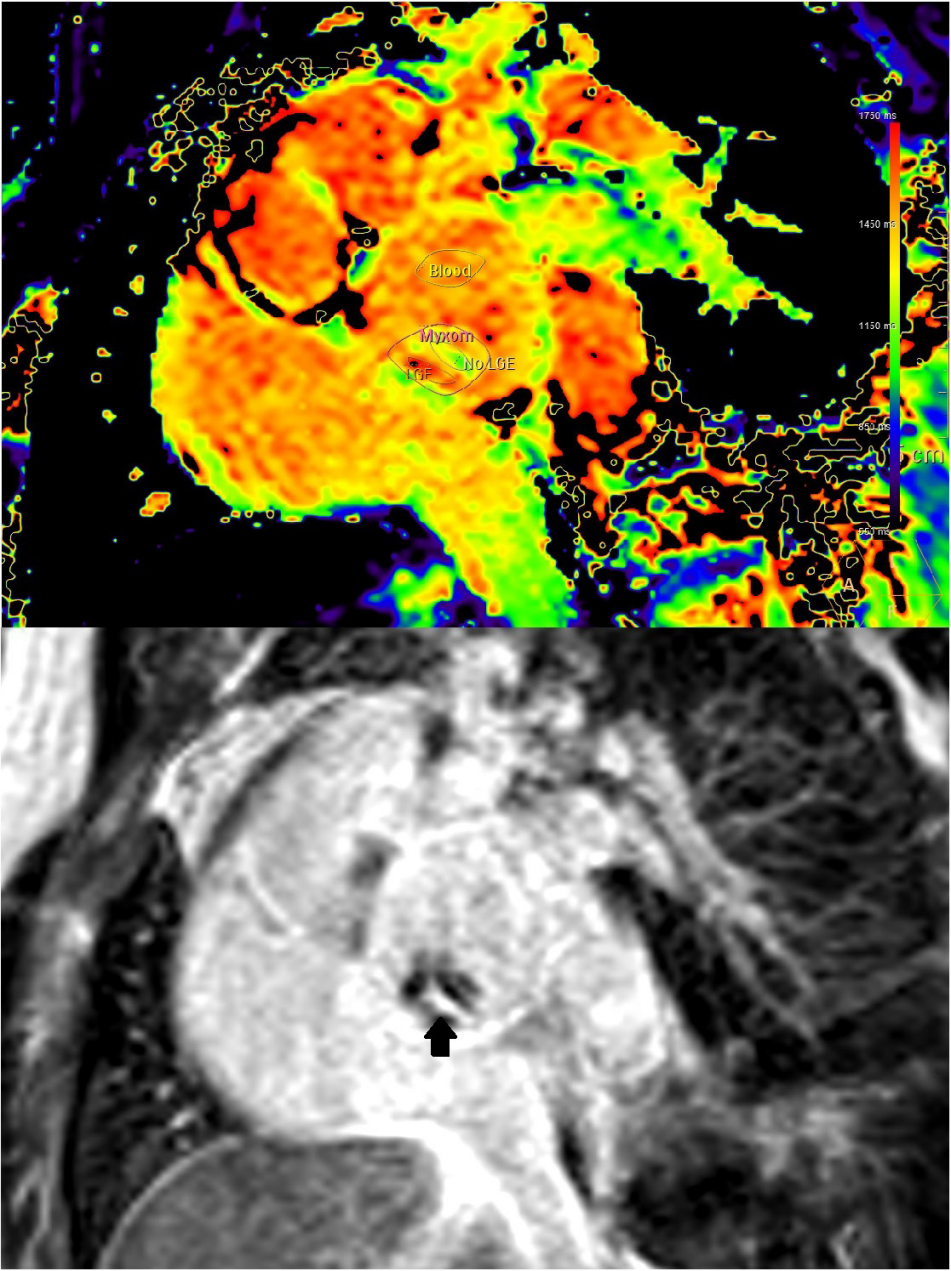
(Case 1) Co-registration of LGE (lower image) and T1-mapping (upper image). Presence of LGE is defined as bright signal on LGE images (arrow). Where areas with and without LGE were discernible on mapping images of the same orientation, respective regions of interest where placed for differential measurements.

Commercially available MR analysis software was used for the Analysis of T1 and T2 relaxation times and the ECV (IntelliSpace Portal Version 11.1, Philips Medical Systems, Best, The Netherlands). ECV was calculated from pre- and post-contrast T1 relaxation times and hematocrit as described previously (9). Hematocrit was measured at the time of MRI using point of care blood gas analysis. In case of visible motion, the motion correction feature was used. The myxoma measurements covered all of the myxoma visible in the respective slice. The myocardial measurements were performed preferentially in the basal septum and preferentially in the same plane as the myxoma. When the basal septum was not visualized in the myxoma imaging plane, a basal short axis plane was used. When the basal septum was not visualized in any plane, a different basal part of the myocardium was measured. When LGE was present inside the myxoma and there was sufficient overlap between LGE and mapping imaging planes, additional mapping measurements were performed in representative areas with and without LGE (Figure 2).

### Histopathology

The excised cardiac masses were received in 4% buffered formaldehyde. The macroscopical findings were photo-documented and the mass was bi-halved or serially sectioned across its base attached to atrial myocardium, as appropriate. At least one, preferably full-thickness tissue section of the tumor was submitted for further histopathological evaluation, which had as a minimum to include the base of the excised mass attached to atrial myocardium, and in addition any suspicious features for malignancy as solid areas or areas of bleeding. None of the lesions showed areas of necrosis.

**Figure 3:**
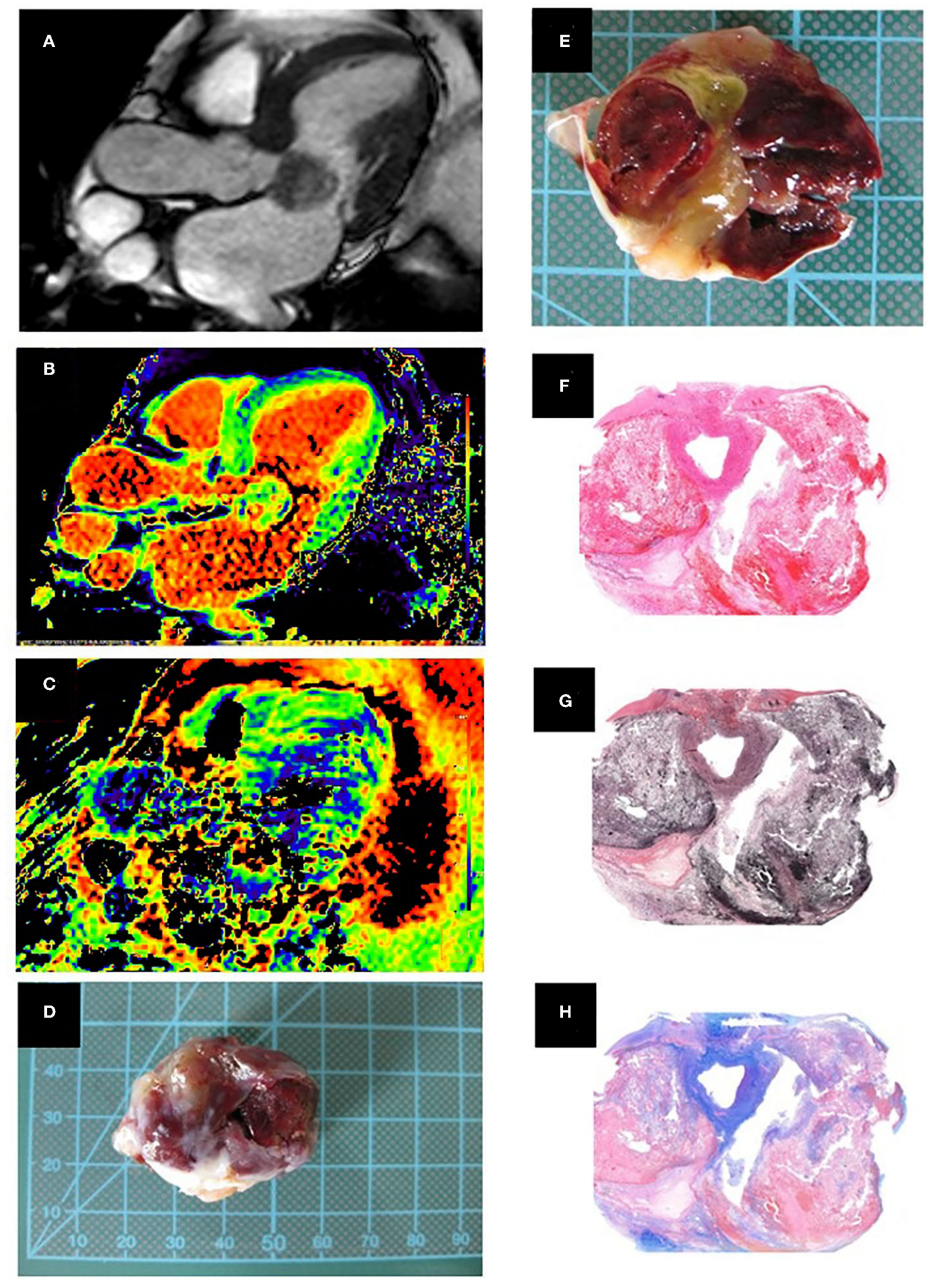
(Case 3). **(A)** Cine image, three chamber view showing the cardiac myxoma attached to the anterior mitral leaflet where the inhomogeneous nature can be appreciated. **(B)** Native T1 relaxation time mapping **(C)** T2 relaxation time mapping. **(D)** Macroscopic appearance of the smooth surfaced resected mass. **(E)** Cut surface of the mass with old and recent hemorrhages and cystic changes in the central portion. Histopathology, low magnification. **(F)** Hematoxylin/eosin stained section shows areas of hemorrhage (red) and cystic change within the cardiac myxoma. **(G)** Elastica van Gieson highlights the fibrous stroma in brown. **(H)** Alcian blue stain highlights myxoid matrix in blue. Histopathological examination showed a well-circumscribed mass with a hemorrhagic and gelatinous cut surface, composed of scattered stellate cells without significant nuclear atypia, which accumulate around vessels, set in a well-vascularized myxoid and fibrous stroma.

The histological evaluation of the atrial masses was performed on 3-micron-thick formalin-fixed paraffin-embedded tissue sections (Figure 3). The first tissue section of each block was mounted on one slide and stained with hematoxylin/eosin. Each of the following sections was stained for collagen (Sirius red) and mucopolysaccharides (Alcian blue/Periodic Acid Schiff Reaction, AB/PAS).

Quantitative histological analysis was performed using a standardized semiautomatic image analysis software (NIS elements AR 4.10.02, Nikon Corporation, Tokyo, Japan). Images of 12 randomly chosen consecutive high power fields (x200 magnification) of the Sirius red stained tissue sections, equaling 1 mm^2^, were obtained with a NIKON Eclipse light projection microscope. Areas of interception between atrial myocardium and atrial mass were due to the overtly high number of large caliber muscular vessels, associated perivascular and interstitial fibrosis avoided, as well as areas of bleeding, pseudocyst formation and areas containing Gamna Gandy bodies (calcifications) throughout the entire lesion. Subsequent image analysis was performed to determine the level of fibrosis, regardless of the staining intensity indicative of the arrangement of the collagen fibers (dark red shades were indicative of established and light red shades were indicative of newly formed fibrosis). The level of interstitial fibrosis was calculated as collagen volume fraction (%) per square millimeter (overall fibrosis).

The level of mucopolysaccharides was assessed in an analogous manner, with the percentage of blue stained background in 12 randomly chosen fields assessed semi-quantitatively with the above mentioned image analysis software.

### Statistical Analysis

Statistical analysis was performed using SPSS for Windows (version 25, SPSS Inc., Chicago, Illinois). All parameters are given as mean ± standard deviation and median. Categorical data are summarized as frequencies and percentages. *T*-tests for dependent samples were performed to evaluate differences for statistical significance. A two-sided *p*-value < 0.05 was regarded as statistically significant. A Pearson correlation analysis was performed for MR parameters (T1, T2, and ECV) vs. histological parameters (fibrosis and mucopolysaccharide volume).

## Results

Patient characteristics and clinical presentation are given in Table 1. Nine patients with histologically confirmed cardiac myxomas and a completed CMR study were included. In one patient, T2 mapping could not be evaluated due to small tumor size and atrial fibrillation.

**Table 1:** Patient's characteristics.

**Case**	**Age (decade)**	**HTN**	**DM**	**IHD**	**TIA/CVS/ embolic event**	**Symptoms**	**Comments**
1	60+	✓	_	_	_	Dyspnea NYHA IV, cough	
2	40+	_	_	_	_	Palpitations, chest tightness	Supraventricular tachycardia, hypothyroidism
3	60+	_	_	_	_	Dyspnea NYHA II-III	
4	60+	✓	_	_	✓	Dyspnea NYHA III-IV, cough	Cerebral small infarcts on CT
5	70+	✓	_	✓	_	Asymptomatic, known since 2011	
6	70+	✓	_	_	_	Dyspnea, palpitations	
7	70+	_	_	✓	_	Asymptomatic	
8	70+	✓	_	_	_	Asymptomatic	Atrial fibrillation, emphysema
9	70+	_	_	_	_	Dyspnea	Dilated cardiomyopathy, dyslipidemia

*HTN, Hypertension; DM, Diabetes mellitus; IHD, Ischemic heart disease; TIA, Transient ischemic attack; CVS, Cerebrovascular stroke; NYHA, New York Heart Association classification. Age categorized in decades for patient confidentiality*.

### CMR

**Figure 4:**
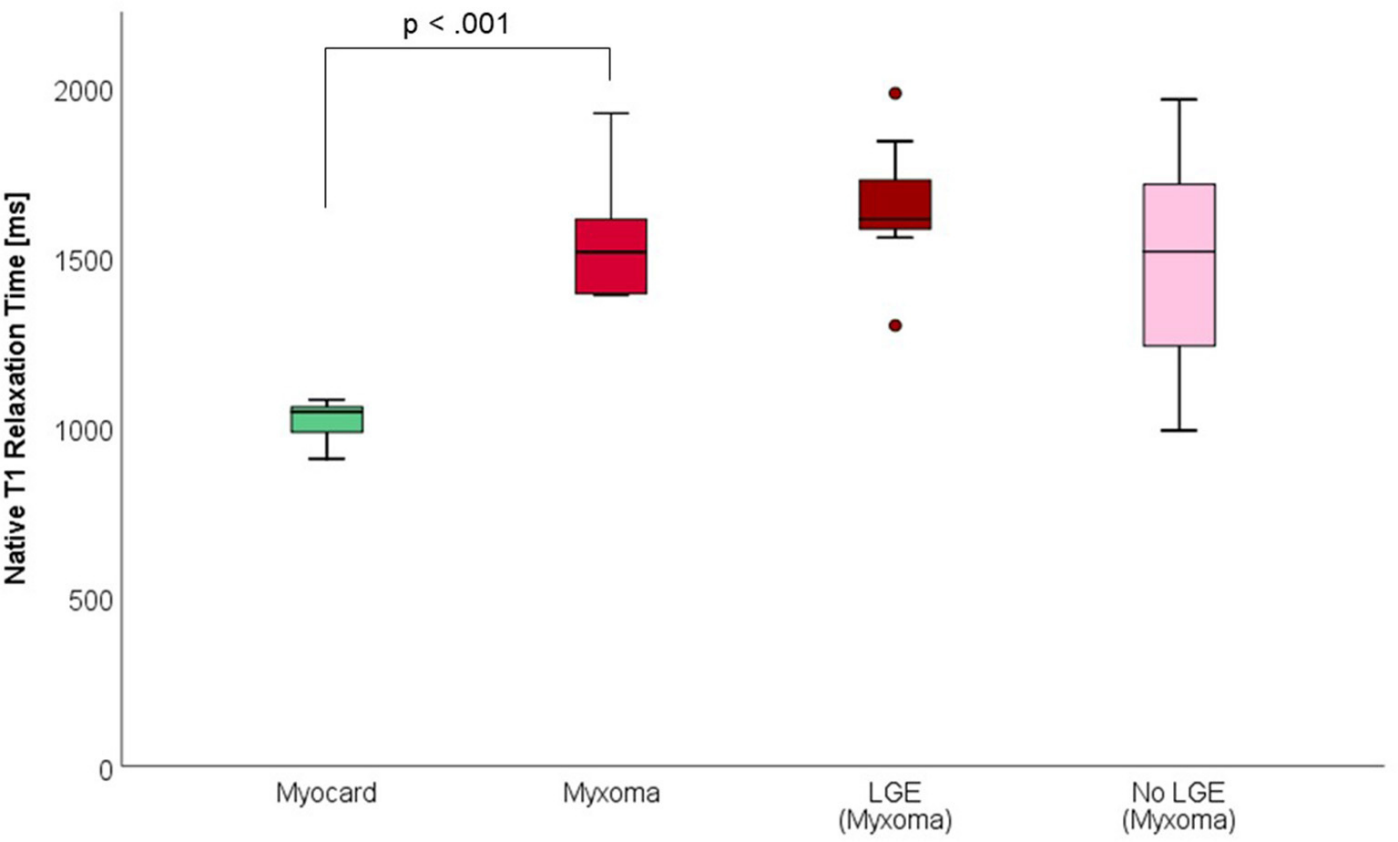
Native T1 Relaxation Times (in milliseconds) of myocardium and myxoma. Where areas with and without LGE were discernible, respective measurements were taken.

**Figure 5:**
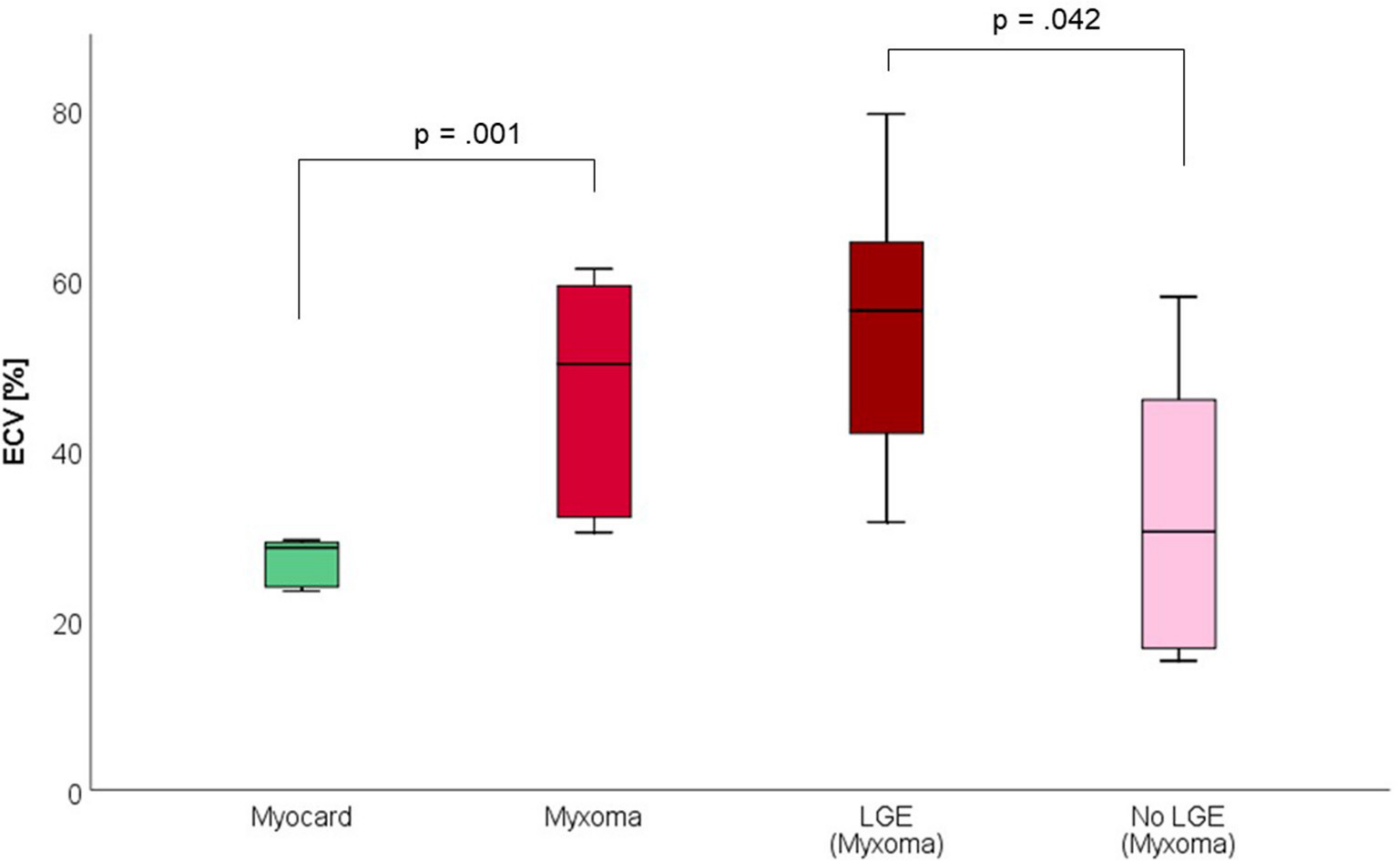
Extracellular volume fraction (ECV, in %) of myocardium and myxoma. Where areas with and without LGE were discernible, respective measurements were taken.

**Figure 6:**
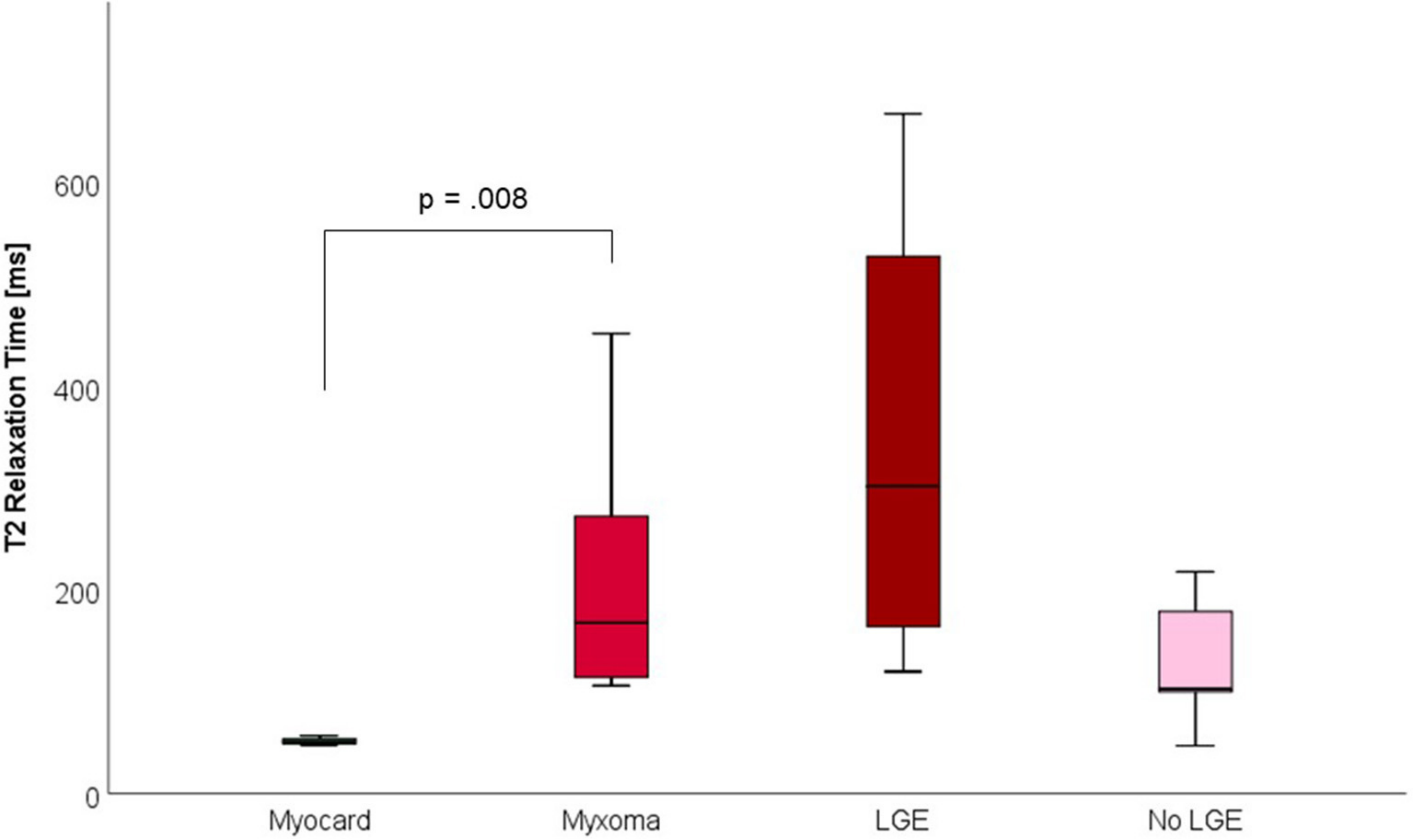
T2 Relaxation Times (in milliseconds) of myocardium and myxoma. Where areas with and without LGE were discernible, respective measurements were taken.

CMR-characteristics of the myxomas are given in Table 2. Cine images confirmed echocardiographic morphological features. Myxomas varied in size between 8 × 8 mm^2^ and 48 × 35 mm^2^. Mobility was variable depending on the thickness and length of the stalk connecting the tumor to the left atrium. CMR showed iso-intense signal of the mass in comparison to the myocardium on T1-weighted images and hyper-intense signal on T2-weighted images in all but one case. The masses did not show signal drop out on fat suppression images. First pass perfusion imaging showed evidence of delayed perfusion in one patient. Late gadolinium enhancement was inhomogeneous in cardiac myxomas in all patients. Compared to myocardium, cardiac myxomas demonstrated higher native T1 relaxation times (1,554 ± 192 ms vs. 1,017 ± 58 ms, *p* < 0.001), ECV (46.9 ± 13.0% vs. 27.1 ± 2.6%, *p* = 0.001), and T2 relaxation times (209 ± 120 ms vs. 52 ± 3 ms, *p* = 0.008) (Tables 3–5, Figures 4–6). Areas with LGE showed higher ECV than areas without (54.3 ± 17.8% vs. 32.7 ± 18.6%, *p* = 0.042), with differences in native T1 relaxation times (1,644 ± 217 ms vs. 1,482 ± 351 ms, *p* = 0.291) and T2 relaxation times (356 ± 236 ms vs. 129 ± 68 ms, *p* = 0.155) not reaching statistical significance.

**Table 2:** Features of cardiac myxomas on cardiac magnetic resonance examination.

**Case**	**Morphology**	**Surface**	**Infil-tration**	**Location**	**SSFP**	**T2-weighted TSE**	**T1-weighted TSE**	**Perfusion**	**LGE**	**Other findings**
1	29 × 26 mm, short stalk		None	Left atrium, lower IAS above AML	Highly mobile, prolapsing in LV cavity	Hyper-intense	Iso-intense	No	Inhomogeneous	
2	20 × 20 mm, round, short stalk	Smooth	None	Left atrium, IAS	Limited mobility, not prolapsing in LV cavity	Hyper-intense	Iso-intense	Delayed local perfusion	Inhomogeneous	
3	15 × 10 mm, long narrow stalk		None	Left atrium, IAS	Highly mobile, not prolapsing in LV cavity	Hyper-intense	Iso-intense	No	Inhomogeneous	
4	43 × 13 mm, elongated, narrow long stalk,		None	Left atrium, IAS	Highly mobile, prolapsing in LV cavity	Hyper-intense	Iso-intense	No	Inhomogeneous	
5	16 × 12 mm, round, broad based	Smooth	None	Left atrium, mid IAS	Limited mobility, not prolapsing in LV	Hyper-intense	Iso-intense	No	Inhomogeneous	Pericardial effusion
6	48 × 35 mm, short stalk		None	Left atrium, IAS	Limited mobility, not prolapsing in LV	Hyper-intense	Iso-intense	Delayed local perfusion	Inhomogeneous	
7	33 × 31 mm, short stalk		None	Left atrium, IAS	Mobile, not prolapsing in LV	Hyper-intense	Iso-intense	No	Inhomogeneous	
8	8 × 8 mm, short stalk		None	Left atrium, IAS	Mobile, not prolapsing in LV	Could not be assessed due to atrial fibrillation	Could not be assessed due to atrial fibrillation	No	No	Atrial fibrillation
9	17 × 12 mm, short stalk		None	Left atrium, IAS	Mobile, not prolapsing in LV	Hyper-intense	Iso-intense	No	Inhomogeneous	Dilated cardiomyopathy

*IAS, Interatrial septum; AML, Anterior mitral leaflet; SSFP, Steady state free precession; TSE, Turbo spin echo sequence; LGE, Late gadolinium enhancement*.

**Table 3:** Native T1 relaxation times [ms].

	**Myocard**	**Myxoma**	**LGE (Myxoma)**	**No LGE (Myxoma)**
Case 1	1,058	1,411	1,612	1,250
Case 2	953	1,577	-	-
Case 3	1,018	1,392	1,841	989
Case 4	905	1,514	1,557	1,516
Case 5	984	1,611	1,611	1,578
Case 6	1,044	1,923	1,982	1,850
Case 7	1,060	1,781	1,608	1,964
Case 8	1,056	1,387	-	-
Case 9	1,079	1,388	1,298	1,226
*N*	9	9	7	7
Median	1,044	1,514	1,611	1,516
Mean	1,017	1,554	1,644	1,482
SD	58	192	217	351

*Case 2: Myxoma area on LGE and Mapping images not overlapping. Case 8: No LGE. LGE, Late Gadolinium Enhancement. N, Number of observations. SD, Standard Deviation*.

**Table 4:** Extracellular volume (ECV) [%].

	**Myocard**	**Myxoma**	**LGE (Myxoma)**	**No LGE (Myxoma)**
Case 1	28.5	59.3	60.7	57.2
Case 2	29.4	60.0	-	-
Case 3	29.1	45.1	52.3	16.6
Case 4	23.4	30.3	31.5	16.8
Case 5	23.9	61.3	68.1	58.0
Case 6	29.0	50.1	79.5	15.2
Case 7	23.9	30.4	31.6	30.4
Case 8	29.3	32.1	-	-
Case 9	27.5	53.1	56.4	34.6
*N*	9	9	7	7
Median	28.5	50.1	56.4	30.4
Mean	27.1	46.9	54.3	32.7
SD	2.6	13.0	17.8	18.6

*Case 2: Myxoma area on LGE and Mapping images not overlapping. Case 8: No LGE. LGE, Late Gadolinium Enhancement. N, Number of observations. SD, Standard Deviation*.

**Table 5:** T2 Relaxation times [ms].

	**Myocard**	**Myxoma**	**LGE (Myxoma)**	**No LGE (Myxoma)**
Case 1	50	109	164	100
Case 2	48	239	-	-
Case 3	52	306	668	47
Case 4	57	157	-	-
Case 5	48	179	302	179
Case 6	56	120	120	218
Case 7	51	452	528	103
Case 8	-	-	-	-
Case 9	51	106	-	-
*N*	8	8	5	5
Median	51	168	302	103
Mean	52	209	356	129
SD	3	120	236	68

*Cases 2, 4, 9: Myxoma area on LGE and Mapping images not overlapping. Case 8: No LGE. LGE, Late Gadolinium Enhancement. N, Number of observations. SD, Standard Deviation*.

### Surgery and Histopathology

All patients underwent surgery for mass excision. Histopathology confirmed nine masses to be myxomas. Macroscopically they had a smooth surface and gelatinous, variably hemorrhagic cut surface. Histologically, there were hypo-cellular myxoid masses, composed of stellate-shaped cells without significant nuclear atypia accumulating around blood vessels. There was no infiltrative growth pattern into the atrial myocardium. Furthermore, there were no atypical mitoses and immunohistochemistry for calretinin showed a diffuse and strong staining reaction of the myxoma cells. Interstitial bleeding and associated hemosiderin-laden macrophages and calcifications were common features. Semi-quantitatively assessed percentage of fibrosis was variable within and between myxomas ranging from 5 to 65% (Table 6). The same applied to mucopolysaccharide content, with the mean percentage per myxoma ranging from 6 to 52%. No significant correlation was found between quantitative CMR and histological parameters (Table 7).

**Table 6:** Histopathological measurements.

	**Mucopolysaccharide area [%]**	**Collagen area [%]**	**Sum [%]**
Case 1	31	5	36
Case 2	52	17	69
Case 3	17	36	53
Case 4	22	16	38
Case 5	6	25	31
Case 6	29	31	60
Case 7	19	22	41
Case 8	6	65	71
Case 9	34	50	84
*N*	9	9	9
Median	22	25	53
Mean	24	30	54
SD	15	18	18

*N, Number of observations. SD, Standard Deviation*.

**Table 7:** Correlations between CMR and histopathological measurements of myxomas.

		**Mucopolysaccharide area [%]**	**Collagen area[%]**
T1 [ms]	Pearson *R*	0.085	−0.282
	Significance (2-sided)	0.829	0.461
	*N*	9	9
ECV [%]	Pearson *R*	0.442	−0.306
	Significance (2-sided)	0.233	0.423
	*N*	9	9
T2 [ms]	Pearson *R*	−0.223	−0.047
	Significance (2-sided)	0.596	0.913
	*N*	8	8

*N, Number of observations. SD, Standard Deviation*.

## Discussion

### Main Findings

To our knowledge, this is the first report to systematically assess T1 and T2 relaxation times in cardiac myxomas. Our study examined parametric CMR-mapping as an addition to standard cardiac tumor imaging protocols for the non-invasive tissue-characterization of myxomas.

We found the following:

- Native T1 and T2 relaxation times and ECV were higher in myxomas compared to the myocardium.
- Within the myxomas, areas with LGE had higher ECV, T1 and T2 relaxation times than areas without, albeit this did not reach statistical significance for T1 and T2 relaxation times due to the small size of the sub-sample.

### Context of Previous Research

So far, only case reports of parametric mapping in cardiac myxomas are published with various CMR protocols. Case 1 of our study, showing elevated T1 and T2 relaxation times and ECV in a left atrial myxoma compared to myocardium, has been previously published as a case report (13). A retrospective analysis of T1 and T2 relaxation times in cardiac tumors on a 1.5 Siemens scanner that included two myxomas showed similar elevations in T1 relaxation times (1346 and 1285 ms) and T2 relaxation times (81 and 76 ms) compared to our study (11). Another observational study of 30 patients with cardiac tumors on a 1.5 Siemens scanner including four myxomas showed T1 relaxation times of 1,682 ± 306 ms (12). In our study, T2 relaxation times were higher in myxomas than in myocardium, which is most likely attributable to the higher fluid content. The T2 relaxation time of water is higher than that of tissue and elevated T2 relaxation times have been found in myocardial inflammation and edema of various etiologies. Differences between normal and inflamed myocardium of 11.7 ms in case of myocarditis and 12 ms in case of Tako-Tsubo cardiomyopathy have been described (6). In our study, there was a mean difference of 117 ms, representing the tissue difference between myocardium and myxomas and not the mere excess of fluid or inflammation.

Myxomas are characterized by a mixed tissue composition, consisting mostly of myxoid stroma that may contain cysts and areas of hemorrhage (15). Compared to myocardium, myxomas appear hyperintense on T2 weighted and mostly isointense on T1 weighted imaging; consequently, T1 and T2 weighted imaging are part of standard cardiac tumor imaging protocols (16, 17). Our findings explain the hyperintense signal of myxomas on T2 weighted images, as tissues with long T2 relaxation times appear bright on T2 weighted sequences. Higher T1 relaxation times lead to lower signal on T1 weighted sequences. The difference in T1 relaxation times found in our study might be too small to lead to visibly detectable differences on T1 weighted sequences, hence the impression of iso-intensity. Our findings are supported by measurements in other tissues that generally show simultaneously elevated T1 and T2 relaxation times in areas with increased fluid content (18).

Late gadolinium enhancement (LGE) is very robust in the identification of focal areas of fibrosis and necrosis. LGE was present in all but one patient with a very small myxoma. LGE was heterogeneous for all cases, indicating the heterogeneous composition of myxomas.

Global alterations of the myocardium are more readily diagnosed by ECV measurements, allowing for the detection of subtle myocardial changes (19). It has been well-correlated to histopathology and collagen content. In a study by Doltra et al., ECV measurements were able to identify reductions in fibrosis in patients with resistant hypertension after renal denervation (20). The elevated ECV of myxomas compared to myocardium reflects the increased extracellular matrix, with a median total histopathological extracellular content (mucopolysaccharide + fibrosis) of 53%, compared to a median MR-derived ECV of 50%. Of note, individual CMR-measurements did not correlate to the histopathological measurements of the same patient. This might be attributable to the heterogeneous tissue composition of myxomas, causing a large intra-subject variation of histological measurements depending on the sampling site, while ECV-measurements provide the average value of a 2 × 2 × 10 mm^3^ voxel.

The statistically non-significant difference in parametric mapping values between myxoma regions with and without LGE warrants further examination in a larger sample. If confirmed, this would underline the heterogeneous nature of myxomas and might improve further tissue characterization.

Mapping of T1 and T2 relaxation times and ECV therefore provides objective, quantitative parameters for the MR-characterization of cardiac myxomas in addition to the visual comparison of unit-less signal intensities on conventional T1 and T2 weighted sequences. While our study does not include other cardiac masses, other studies have provided case reports (Table 8) (11, 12).

**Table 8:** Relaxation times in cardiac masses compared to myocardium.

**Tissue**	**Native T1 relaxation time**	**T2 relaxation time**
Myxoma	↑	↑
Thrombus	↔	↑
Fibroelastoma	↑↑	↑↑
Calcification	↓↓	(↓)
Lipoma	↓↓	↑
Melanoma	↓	↔
Renal cell carcinoma	↑	↑
Hemangioma	↑	↑
Rhabdomyoma	↔	(↑)

*Myxoma data from our study. Other data based on published case reports (11, 12). Up-arrows denote increased, down-arrows decreased relaxation time compared to myocardium*.

### Limitations

The main limitation of our study is its small sample size. Nevertheless, it is the largest study of parametric mapping in this rare entity.

The high mobility of myxomas makes it difficult to seize them on CMR-images. However, in all of the cases they still could be identified. The current spatial resolution of CMR makes small sized tumors difficult to recognize. The smallest tumor we were able to depict on cine images was 8 × 8 mm in size. More studies are needed to establish a cut-off point for tumor size to be detected my CMR.

T1 and T2 mapping are prone to motion artifacts due to the high mobility as well as arrhythmias. Tumors attached to the mitral valve show the least motion and best visualization when the valve is closed. The feasibility of changing the phase of image acquisition from diastole to systole should be studied and accordingly may facilitate T1 and T2 mapping of cardiac myxomas.

## Conclusion

We demonstrated that native T1 mapping, ECV, and T2 mapping offer an objective and quantitative measurement of tissue alterations seen in conventional T1 and T2 weighted and LGE imaging. We therefore suggest integrating these in the routine evaluation of morphologically suspected cardiac myxomas, adding valuable information.

## Data Availability Statement

The original contributions presented in the study are included in the article/supplementary materials, further inquiries can be directed to the corresponding author.

## Ethics Statement

The studies involving human participants were reviewed and approved by Ethikkommission der Charité Universitätsmedizin Berlin, Germany. Written informed consent for participation was not required for this study in accordance with the national legislation and the institutional requirements.

## Author Contributions

All authors listed have made a substantial, direct and intellectual contribution to the work, and approved it for publication.

## Conflict of Interest

PD owned stock of Siemens AG and Bayer AG. BS was an employee of Philips Healthcare. BP had received advisory board and lecture honoraria from Bayer Healthcare, Novartis, Merck Sharp and Dohme, Stealth Peptides, Sanofi, and Servier. KW had received lecture honoraria from Roche. SK was supported by Philips Health Care. The remaining authors declare that the research was conducted in the absence of any commercial or financial relationships that could be construed as a potential conflict of interest.
